# The ‘Universal Approach’ for Dupuytrenʼs disease: A safe and reproducible sequence for planning fasciectomy incisions

**DOI:** 10.1016/j.jpra.2024.07.003

**Published:** 2024-07-20

**Authors:** Adam Stoneham, Sherif Fetouh, Muattaz Kazzam, David Warwick

**Affiliations:** a Department of Trauma and Orthopaedics, University of Southampton Teaching Hospital, Tremona Road, Southampton SO16 6YD, United Kingdom; b Department of Trauma and Orthopaedics, North Shore Hospital, Shakespeare Road, Takapuna, Auckland 0620 New Zealand; c Royal National Orthopaedic Hospital, Brockley Hill, Stanmore HA7 4LP, United Kingdom

**Keywords:** Dupuytren's, Fasciectomy, Invision, Z plasty, Skin graft

## Abstract

Dupuytren's disease continues to present many challenges for the surgeon. A variety of surgical approaches and their variations have been described in the literature, further complicated by the degree of skin shortage and/or the need for local flap procedures or a full thickness skin graft. In the face of all these decisions – none of which is supported by Level 1 evidence – it can be very difficult to plan the best incision(s). We describe a safe and reproducible technique to plan fasciectomy incisions in primary or recurrent Dupuytren's disease. Our short communication and accompanying artwork demonstrates the anatomical landmarks and a simple decision-making algorithm based on just 3 key stages: (1) Proximal incision planning and execution of the palmar release(s); (2) Extension distally into the digit(s) based on the tissue quality, with either with zigzag (Brunner's) or a midline longitudinal (McIndoe) incision(s); (3) Flap assisted closure or coverage with a full thickness skin graft where required.

## Introduction

Fasciectomy is an effective treatment for Dupuytren's Disease, either in the primary setting or following failed open or percutaneous procedures.^1^ The surgical approach depends on many factors including site and nature of the cord(s), quality of the skin, and severity of the contracture. This is further complicated by the degree of skin shortage and the need to perform local flap procedures or a full thickness skin graft (FTSG).^2^

Many techniques and modifications have been described: Bruner's, (zig zag), Jacobsen's (L-shaped), McIndoe (midline longitudinal), McCash (open palm) amongst others.^3^ In the face of all these decisions – none of which is substantiated by Level 1 evidence – it can be difficult to plan a consistently safe and reliable pattern of surgical incisions, especially for surgeons with less experience. We therefore conceived a simple, step-wise technique which we find to be adaptable to most cases.

### Step 1

**Figure 1:**
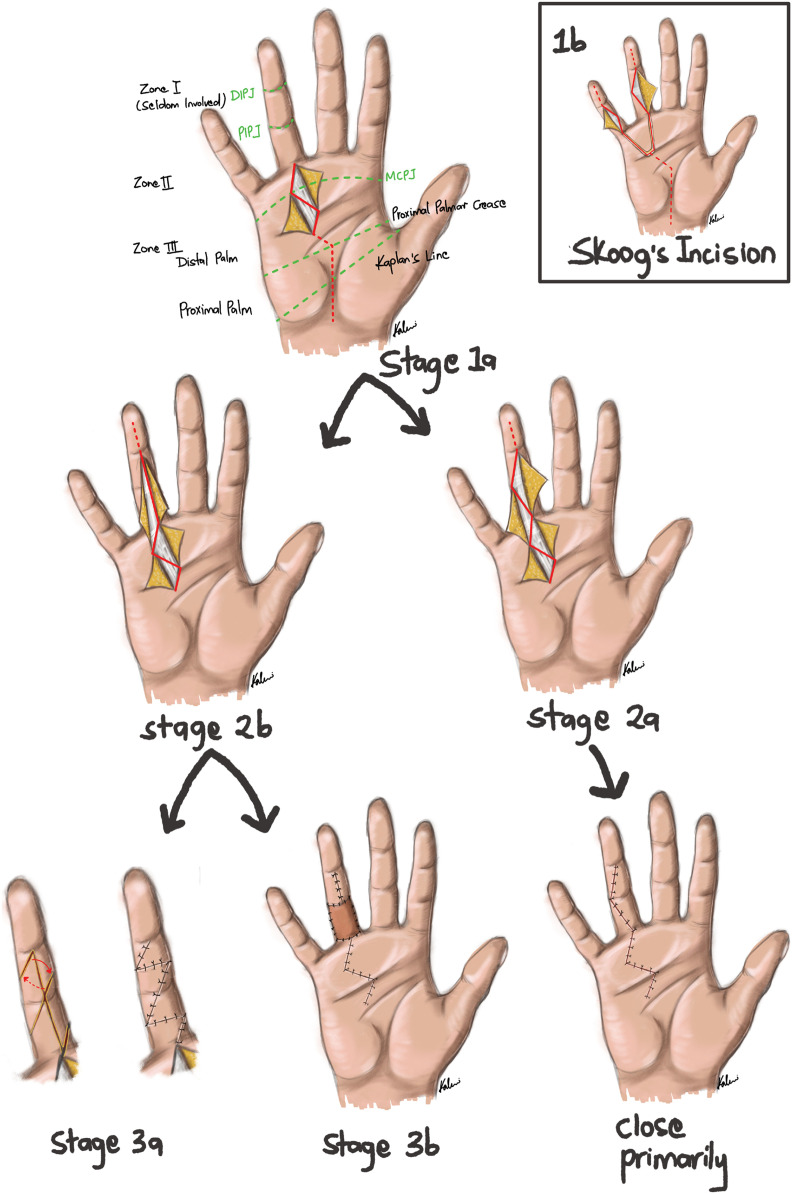
The ‘Universal Approach’: a simple algorithm for incision planning in Dupuytrens surgery. (1a) Mark out a zig zag incision in the palm. Adjacent digits can be accommodated by extending the incision transversely (after Skoog) (1b). (2) Decide if the digital skin is of sufficient area and quality to perform oblique (Brunner's) incisions [2a] or if in doubt make a midline longitudinal incision [2b] (after McIndoe). (3) A midline incision can either be closed using one or more Z plasties [3a] or alternatively excised entirely (dermofasciectomy) and covered with a full thickness skin graft [3b].

Start in the palm. Mark out a chevron or zig zag incision over the diseased tissue [Figure 1: Stage 1a]. Images show markings on the ring finger but it is equally applicable to the other digits. A transverse incision (after Skoog) can be extended laterally in the case of adjacent digits [Figure 1: Stage 1b].

Identifying the neurovascular bundles is easier and safer proximal to the transverse palmar ligament where they are separated from the flexor tendons by the septa of Legueu & Juvara (which run deep to superficial).

Correcting the proximal deformity at the metacarpal phalangeal joint (MCPJ) will determine the amount of distal dissection required and help to get the angle of subsequent incisions correct (ideally 45° to the longitudinal axis of the digit). Sometimes contractures are ‘dynamic’ i.e. they cross adjacent segments and releasing them proximally at the MCPJ gains ‘free’ distal extension at the PIPJ.

In the little finger, release the abductor cord, which connects the abductor digiti minimi to the contracted lateral digital sheet. This simple manoeuvre will often greatly improve the proximal interphalangeal joint (PIPJ) contracture and render distal neurovascular dissection much easier.^4^

### Step 2

Having achieved maximal release in the palm, decide whether the skin overlying the proximal phalanx is healthy or compromised by disease or scar tissue.

• Healthy skin / lesser contractures suit Bruner's incisions (oblique incisions between the flexion creases) or modified Bruner's incisions (oblique incisions only as far as the midline) which are closable by direct suture and familiar to most surgeons. [Stage 2a]. Consider small V-Y flaps at the apices if required.
• Poor quality / contracted skin is best addressed with a single midline incision (as advocated by McIndoe) from the MCP crease to the distal interphalangeal (DIP) crease. [Stage 2b].

### Step 3

Where a midline longitudinal incision has been used, the final decision is whether to lengthen the scar using one or more Z plasties [3a] or to excise completely and cover with a full thickness skin graft [3b], the latter being a good option for skin of poor quality or of inadequate surface area.

• Design 1 or 2 Z plasties between the MCP and PIP creases and/or the PIP and DIP creases depending on the lengthening required (60° at the apices delivers 75 % increased length). Mark out the 2 limbs of the flap at equal lengths and parallel to each other as shown.
• Take the FTSG from the upper inner arm, inset with 5/0 Vicryl Rapide, and tie over a wool-paraffin pad. To avoid compromising the inosculation of the graft take care not to disturb the flexor tendon sheath or to damage the paratenon during dissection.

## Conclusion

The ‘Universal Approach’ is safe, reproducible, and adaptable to almost all primary and revision cases. It is a useful tool to teach surgeons-in-training as it provides a reproducible sequence of incisions which can be adapted depending on how the case evolves.

## Funding

This work received no external funding.

## Informed consent

No human subjects were investigated for this report.

## Ethical approval

No ethical approval was required.

## CRediT authorship contribution statement

**Adam Stoneham:** Writing – original draft, Writing – review & editing. **Sherif Fetouh:** Writing – review & editing. **Muattaz Kazzam:** Writing – review & editing. **David Warwick:** Conceptualization, Supervision, Writing – review & editing.

## Declaration of competing interest

No authors, their immediate family, and any research foundation with which they are affiliated received any financial payments or other benefits from any commercial entity related to the subject of this article.
